# Selection and Evaluation of Reference Genes for Expression Studies with Quantitative PCR in the Model Fungus *Neurospora crassa* under Different Environmental Conditions in Continuous Culture

**DOI:** 10.1371/journal.pone.0112706

**Published:** 2014-12-04

**Authors:** Kathleen D. Cusick, Lisa A. Fitzgerald, Russell K. Pirlo, Allison L. Cockrell, Emily R. Petersen, Justin C. Biffinger

**Affiliations:** 1 National Research Council Associateship, U.S. Naval Research Laboratory, Washington, District of Columbia, United States of America; 2 Chemistry Division, U.S. Naval Research Laboratory, Washington, District of Columbia, United States of America; Friedrich Schiller University, Germany

## Abstract

*Neurospora crassa* has served as a model organism for studying circadian pathways and more recently has gained attention in the biofuel industry due to its enhanced capacity for cellulase production. However, in order to optimize *N.* crassa for biotechnological applications, metabolic pathways during growth under different environmental conditions must be addressed. Reverse-transcription quantitative PCR (RT-qPCR) is a technique that provides a high-throughput platform from which to measure the expression of a large set of genes over time. The selection of a suitable reference gene is critical for gene expression studies using relative quantification, as this strategy is based on normalization of target gene expression to a reference gene whose expression is stable under the experimental conditions. This study evaluated twelve candidate reference genes for use with *N. crassa* when grown in continuous culture bioreactors under different light and temperature conditions. Based on combined stability values from NormFinder and Best Keeper software packages, the following are the most appropriate reference genes under conditions of: (1) light/dark cycling: *btl*, *asl*, and *vma1*; (2) all-dark growth: *btl*, *tbp*, *vma1*, and *vma2*; (3) temperature flux: *btl*, *vma1*, *act*, and *asl*; (4) all conditions combined: *vma1*, *vma2*, *tbp*, and *btl*. Since *N. crassa* exists as different cell types (uni- or multi-nucleated), expression changes in a subset of the candidate genes was further assessed using absolute quantification. A strong negative correlation was found to exist between ratio and threshold cycle (C_T_) values, demonstrating that C_T_ changes serve as a reliable reflection of transcript, and not gene copy number, fluctuations. The results of this study identified genes that are appropriate for use as reference genes in RT-qPCR studies with *N. crassa* and demonstrated that even with the presence of different cell types, relative quantification is an acceptable method for measuring gene expression changes during growth in bioreactors.

## Introduction

*Neurospora crassa* is a filamentous fungus that has served for nearly 50 years as the model organism for studying circadian pathways and the molecular clock in eukaryotes [1]. It is classified under the phylum Ascomycota and is widely distributed in nature [1], typically found growing on vegetation killed by fire [2]. As such, it is able to synthesize and secrete high levels of all main enzyme types involved in lignocellulosic biomass degradation and is also able to convert pentose sugars, cellulose polymers, and agro-industrial residues to ethanol [3]. Its genome is predicted to contain twice as many cellulase genes in comparison the current state-of-the-art industrial strain used in biofuel production, *Trichoderma reesei* [3]. Additionally, recent work has shown that the variety of molecular, genetic, and biochemical techniques available for *N. crassa* can advance the analyses of fungal deconstruction of plant biomass [4]. Therefore, great interest in the use of *N. crassa* for biotechnological applications has sparked in recent years.

Gene expression profiling is an effective means of studying an organism’s response to its environment, a necessary component in identifying the genes and regulatory mechanisms associated with biological processes in *N. crassa* such as circadian pathways and important biotechnological applications such as cellulase degradation. Recent work in our lab has utilized *N. crassa* maintained in continuous, long-term bioreactor cultures in an effort to understand the triggers of cellulase production. Most *N. crassa* studies are based on either racetube or batch culture conditions. Understanding the complexity of *N. crassa* growth in bioreactors is lacking and more importantly, the identification of optimal reference genes for molecular analysis needs to be addressed.

Reverse-transcription quantitative PCR (RT-qPCR) is a high-throughput means of studying the expression of a large suite of genes over time and under different conditions. With this technique, two quantification strategies are available to measure the level of expressed genes: absolute and relative quantification. Absolute quantification relates the fluorescent PCR signal of the sample to input copy number using an external calibration curve, while relative quantification measures the relative change in mRNA expression levels [5]. Relative expression is based on normalization of target gene expression to an internal control gene, often referred to as a “reference” or “housekeeping” gene, that should not vary in the cells under investigation or in response to environmental or experimental conditions [6]. The selection of a suitable reference gene is critical for gene expression studies using relative quantification [5]. However, these reference genes, thought to be constitutively expressed and minimally regulated, are commonly used for expression profiling in RT-qPCR assays without *a priori* conformation that mRNA levels do not change under the experimental conditions being investigated. The use of reference genes necessitates that they be validated for the specific experimental set-up and it is typically necessary to choose more than one [7]. Work by Vandesompele *et al* [6] demonstrated that the typical procedure in which a single reference gene is used for normalization can lead to relatively large errors [6], and it is now common to employ a combination of genes. Thus, in order to understand the metabolic processes when *N. crassa* is grown under different environmental conditions, the selection and evaluation of reference genes must be addressed.

**Figure 1:**
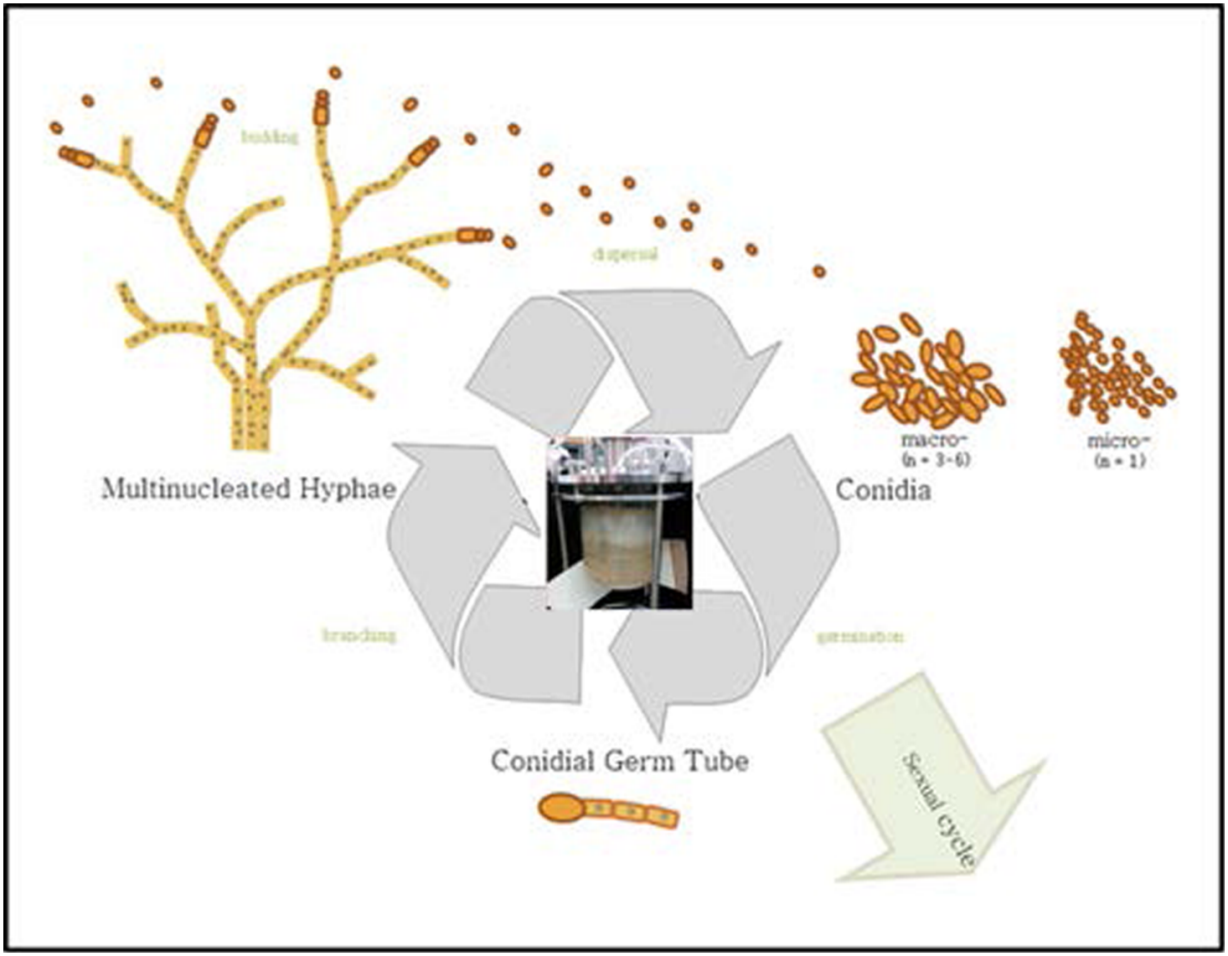
Generalized lifestyle of *N. crassa* during which different cell types occur. Center image illustrates bioreactor set-up. Within the bioreactors utilized in this study, *N. crassa* was present as both multi- and uninucleated cells.

*N. crassa* is a morphologically complex multicellular organism, possessing at least 28 distinct cell types [8] between the vegetative (asexual) and sexual phases. In bioreactors, it is maintained in the vegetative phase. The primary cells occurring in the vegetative phase include hyphae, macro- and microconidia, all of which are comprised of several different types [8]. One of the biological differences among these cell types is the number of nuclei per cell: the macroconidia are multinucleated, possessing on average between 3–6 nuclei per cell, while microconidia possess only a single nuclei (Figure 1). This life cycle trait must be taken into account when performing gene expression studies, particularly under continuous, long-term culture conditions such as those conducted in bioreactors. In general, our bioreactors are dominated by clusters of aggregated colonies dispersed throughout the vessel, similar in cell type composition to the mats observed in batch cultures yet comprised of more discrete units. Therefore, it is necessary to determine whether changes in gene expression are the result of true fluctuations in transcripts, or whether an increase or decrease in transcripts (i.e. gene expression) is actually a reflection in gene copy number fluctuation, which would occur when cells are a mix of macro- and micro-conidia. One means of assessing gene expression changes is to determine the number of transcripts normalized to a certain value such as cell number, volume of sample, or mass. This number can then be related to the gene copy number normalized to the same units to provide a ratio of the transcript to gene ratio. These data standardize the transcripts to the gene copy number and so determine whether the gene is changing expression, or whether transcript variation is a result of gene variation. This type of situation applies to cultures that occur as multiple cell types during which some morphologies are multinucleated.

While numerous gene expression studies have been performed with *N. crassa*, little to no information is provided as to reference gene validation, and the extent of gene expression fluctuation as a result of changes in gene copy number (i.e. as cells progress from hyphae to macroconidia) has not been addressed. Additionally, genes that are commonly used as reference genes in other organisms have been shown to be under clock control in *N. crassa*. In general, the actin gene is frequently used as a normalizer in gene expression studies [9]–[11], though in most cases there is no mention of stability verification. Therefore, the overall objective of this study was to examine a suite of genes for the potential to serve as appropriate reference genes under a variety of environmental conditions during growth in *N. crassa*. We evaluated a set of 12 genes under different environmental conditions using a combination of both relative and absolute quantification. Using this combination allowed for a more comprehensive assessment of fluctuations in expression, as the transcript copy number per mL was calculated and its ratio to gene copy number determined. Additionally, the well-established software programs BestKeeper [12] and NormFinder [13] were used to determine expression stability and rank of all genes to assess their suitability as reference genes.

## Materials and Methods

### Organism Maintenance

*N. crassa* cultures were maintained on agar slants (1.5% w/v sucrose with Vogel’s medium [14]) at −20°C for up to 1 month. Slants were prepared following standard protocols. Briefly, the medium comprised of Vogel’s salts, 1.5% sucrose, and 1.5% agar was autoclaved and cooled to ≤50°C whereupon the trace minerals and biotin solutions (1.0×10^−5^ M) were added. Agar was distributed among test tubes and cooled at an angle, resulting in a “slanted” agar medium. Strain X-661 (*ras-1bd* mutant with the *frq:luc* transcriptional reporter) was used for these studies.

### Bioreactor culture conditions and cell harvesting

**Figure 2:**
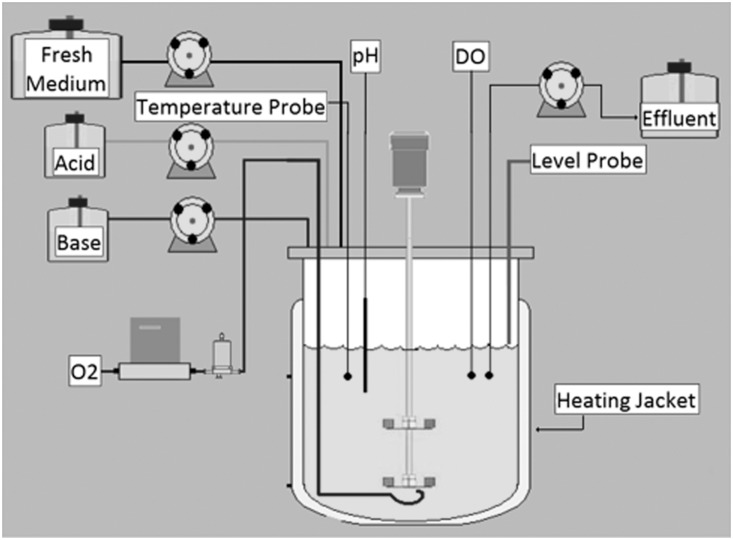
Schematic of bioreactor design and operation.

New slants were inoculated from the frozen stock culture at the start of each experiment. *N. crassa* X-661 grew on slants for 2–3 days at 30°C in constant darkness. Conidia were isolated from slants by washing the slant culture with ∼1 mL of Vogel’s medium [14] lacking a carbon source. The collected material was centrifuged (10,000 rpm, 1 min) and the resulting conidial pellet was washed 3 times with Vogel’s medium (no C-source). The final pellet was re-suspended and inoculated into 60 mL of Vogel’s medium with 1% glucose, 0.2% Junlon, (100,000 MW, Sigma Aldrich) and 0.008% Antifoam 204 (Sigma Aldrich). The culture was grown at 30°C with shaking for 24 hrs with light. This culture was inoculated into a BioFlo/CelliGen 115, 1.3 L bioreactor (Figure 2) containing the same medium composition and grown in batch for 24 hrs with light. Continuous flow into and export from the culture was started as the cells reached the exponential growth phase. When the cells reached steady state (∼11 hrs later) the light remained on for 11 hrs and then was shut off. At this point, the cells were grown in one of three conditions: continuous all-dark conditions, under light/dark cycling, or under temperature flux (25°C/37°C) in constant darkness. The light/dark and temperature flux oscillated with 22-hr periods (11 hrs light, 11 hrs dark) in keeping with the period length of *N. crassa* circadian oscillations. During the experiments, *N. crassa* cultures were maintained in steady-state conditions, with pH 5.5 and 10% dissolved O_2_. The O_2_ was bubbled through the medium at 1.0 L/min. A dynamic agitation program was used to keep the cells suspended in bioreactor cultures. The program controlled the rotation rate of the propeller in the reactor and was set up as a 10-min cycle using the BioCommand Batch Control Plus BioProcessing software (New Brunswick Scientific, #M1326-0051, revision B). The propeller rotated at 1,000 rpm for 1 min, 100 rpm for 1 min, and then 400 rpm for 8 min. This program was started ∼1–2 hrs following bioreactor inoculation. The temperature was either maintained at 25°C or cycled (with 11-hr periods) between 25°C and 37°C. The bioreactors were equipped with an external heating jacket and an internal cooling finger and temperature probe to accurately maintain the temperature. A temperature cycling program (BioCommand software) was used for varied temperature experiments. The growth state of the cells (i.e., log phase, steady-state) was monitored using the CO_2_ output. A CO_2_ gas sensor (Vernier) was used to detect CO_2_ produced by the culture. The CO_2_ data was recorded using the LoggerLite software (version 1.6.1). Samples were removed under darkroom light conditions (safelight) and the 1.5 mL aliquots were flash frozen in centrifuge tubes and stored at −80°C. Biological replicates, in the form of individual bioreactors, were performed for each type of environmental condition, with a total of 8–10 time points collected per condition.

### DNA extraction

Genomic DNA was extracted from biological replicates using the DNeasy Plant Mini kit (Qiagen, Valencia, CA) following the manufacturer’s instructions, with slight modifications. Briefly, cells from 1 mL of a bioreactor sample were disrupted using a mortar and pestle under liquid nitrogen. Following addition of 400 µL Buffer AP1 and 4 µL RNase A, the slurry was ground an additional 1–2 min. The entire volume was then transferred to a 1.5 mL microfuge tube, vortexed vigorously, and 400 µL of this slurry removed for DNA extraction. The remaining steps followed the manufacturer’s protocol. DNA was eluted in nuclease-free water rather than Buffer AE. DNA concentration and purity were assessed using the Nanodrop-2000 (Thermo Fisher Scientific, Wilmington, DE). DNA used in quantitative PCR (qPCR) assays was diluted 1∶5 in nuclease-free water prior to use.

### RNA isolation and cDNA synthesis

Total RNA was extracted frozen from a 1 mL bioreactor sample using the RNeasy Plant Mini kit (Qiagen, Valencia, CA) following the manufacturer’s protocol, with slight modifications. Briefly, cells were disrupted using a mortar and pestle under liquid nitrogen. Four hundred fifty microliters of buffer RLC was added and the slurry mixed until pipetteable. The sample was vortexed vigorously and 450 µL added to the QIAshredder spin column. Subsequent steps followed the manufacturer’s protocol. The DNase digestion was extended to 1 hr. An additional spin at top speed (16,000 rpm) for 1.5 min was included immediately prior to elution in RNase-free water. Total RNA concentration and purity was measured using the Nanodrop-2000.

Total RNA was converted to cDNA using the High-Capacity RNA-to-cDNA kit (Applied Biosystems, Foster City, CA). The protocol was optimized to include less template RNA and a larger overall reaction volume, as preliminary screening with several of the potential reference genes revealed greater fluctuations in C_T_s when using the recommended 2 µg in a 20 µL volume (data not shown). The following protocol was used for all reverse transcriptase reactions: total RNA was first diluted 1∶5 in nuclease-free water, and 500 ng added to the reaction. Each reaction contained: 2 µL 20x enzyme mix, 2x RT buffer, a volume equivalent to 500 ng total RNA, and brought to a final volume of 40 µL with nuclease-free water. Controls consisted of all components except the enzyme, in which case an equal volume of nuclease-free water was added. Reactions were incubated in a 2720 thermocycler (Applied Biosystems, Foster City, CA) under the following conditions: 37°C for 60 min followed by heating to 95°C for 5 min. Prior to use in qPCR, samples were again diluted 1∶5 in nuclease-free water.

### Candidate reference gene primer design and validation

Quantitative PCR assays were developed for a suite of candidate reference genes (Table 1) based on the SYBR Green chemistry [15]. For each assay, primers were designed that targeted an ca. 100–130 bp region of the coding sequence of each gene (Table 1). When possible, primers were designed to span exon-exon boundaries. Both coding and genomic sequences of each gene were obtained from the *N. crassa* database located on the Broad Institute website (http://www.broadinstitute.org/annotation/genome/neurospora/GeneIndex.html). Primers were designed using the Primer3 software (http://bioinfo.ut.ee/primer3/), with an annealing temperature of 60°C. Primers were examined for potential secondary structures using Mfold [16]. The potential for self and cross dimer formation was examined for each of the primer sets using an online oligonucleotide analysis tool (https://www.operon.com/oligos/toolkit.php). Self-complementarity was determined using the oligonucleotide properties calculator (http://www.basic.northwestern.edu/biotools/oligocalc.html). Primers were obtained from Invitrogen (Carlsbad, CA). Primer sets were optimized over concentrations spanning 150–300 nM, with an annealing/extension temperature of 60°C. Product specificity was determined via melt curve analysis. The PCR efficiency of each assay was determined using LinRegPCR [17].

**Table 1:** List of candidate reference genes.

GeneName	Accession #	Description	Primer Sequence(5′-3′)	AmpliconLength	T_M_	PCR *E*	FinalConcentration
*act*	NCU04173	beta-actin	F:GATCGGTATGGGCCAGAAGG	110	79.10	1.91	300 nM
			R:CGTCCCAGTTGGTAACGACA				
*acl*	NCU06783	ATP citrate lyase	F:AGATCCTGATCCCCGTCGACC*	120	80.55	1.92	300 nM
			R:AACCTCGGCAGAGGTGGCATC*				
*adk*	NCU00414	adenosine kinase	F:TCCTCCACGATGCTGTCAAG	148	83.82	1.86	300 nM
			R:TCAAGGTCGTAGTGGTTGGC				
*asl*	NCU09119.7	ATP synthaselyase gamma subunit	F:TACCAACGCCAAGGACATCG	114	81.35	1.89	300 nM
			R:GAGTAGTCGCCCTTGAGCTG				
*btl*	NCU04054	beta-tubulin	F:CCACTTCTTCATGGTCGGCT	103	83.21	1.83	150 nM
			R:CTTGGGGTCGAACATCTGCT				
*frh*	NCU03363	Frq-interactingRNA helicase	F:TAGCAAGAATGCCAGGTGGG	139	82.35	1.92	300 nM
			R:GATCTCGTCAACGGCTTTGC				
*l6*	NCU02707	60S subunitribosomal protein	F:AGAAGGAGGTTTCCAGCAGC	148	82.25	1.90	300 nM
			R:CACTTCATCTCGTGGGGCTT				
*tbp*	NCU044770	TATA binding boxprotein	F:GGTGCCAAGTCCGAAGATGA	136	79.38	1.94	300 nM
			R:TGGGGAACTTGATGTCGCAA				
*trk*	AJ009758.1	K transporter	F:AGTACACTATACCCTCATCATTG	110	78.40	1.89	300 nM
			R:CCCCAGCAGCGAAGAAGAG				
*vma1*	J03955.1	vacuolar ATPase subunit 1	F:CCAACAAAATGGCGCCGAG	111	80.45	1.89	300 nM
			R:CCAACTCATACATAGCAACACC				
*vma2*	J03956.1	vacuolar ATPasesubunit 2	F:GTCGTCCAGGTCTTCGAGG	127	78.90	1.89	150 nM
			R:TGCCGGAACCATCAAAGATACG				
*vma3*	NCU01332.7	vacuolar ATPasesubunit 3	F:ACAGGACCACTACGCTCTCT	114	80.08	1.90	300 nM
			R:ACACCAGCATCACCGACAAT				

*Primers from [25].

### Construction of external standards for absolute quantification

The expression of a subset of the candidate reference genes was also assessed via absolute quantification of the ratio of transcripts to gene copies per mL of sample. This was achieved though the creation of external calibration curves. Plasmid-based standards were constructed for the actin (*act*), ATP synthase lyase γ subumit (*asl*), adenosine kinase (*adk*), and vacuolar ATPase subunit 3 (*vma3*) genes and used for absolute quantification of both transcript and gene copy number. Standards were created via amplification from genomic DNA using gene-specific primers (Table S1). DNA was used rather than RNA as it has been shown that external calibration curves constructed with DNA rather than RNA serve as better models for mRNA quantification due to higher sensitivity, increased quantification range, higher reproducibility, and is more stable than the RNA calibration curve [18]. PCRs were conducted in 25 µL reaction volumes with the PCR Reagent System (Invitrogen, Carlsbad, CA) in a 2720 thermocycler (Applied Biosystems, Foster City, CA) and contained (as final concentration): 1x PCR buffer (minus Mg), 2 mM MgCl_2_, 0.25 mM each dNTP, 200 nM each forward and reverse primer, 0.5 U Taq polymerase, and brought to a final volume of 25 µL with nuclease-free water. Thermocycling conditions consisted of 94°C, 3 min; 35 cycles of 94°C, 45 s, 51°C (48°C for *act*) for 30 s, 72°C, 1 min, and a final extension at 72°C, 7 min. PCR products were visualized by gel electrophoresis to confirm product amplification of the proper size. The resulting amplicons were cloned into the pCR4 vector using the TOPO TA cloning kit (Invitrogen, Carlsbad, CA) and transformed into chemically competent TOP10 *E. coli* following the manufacturer’s instructions. Cells were spread onto Miller’s Luria Broth (LB) plates containing 50 µg mL^−1^ kanamycin (LBkan_50_) and incubated overnight at 37°C. Colonies were screened for inserts of the expected size via the gene-specific PCRs described above. Colonies producing a band of the expected size were grown overnight in 2 mL LBkan_50_ broth. Plasmids were isolated using the QIAprep Spin Miniprep kit (Qiagen, Valencia, CA) and sequenced using the M13 forward and reverse primers by GeneWiz, Inc. (Germantown, MD). Sequence identity was confirmed using the standard nucleotide BLAST function on the NCBI website, with organism specificity set to “Neurospora.” The number of copies per µL was calculated using the equation: X g µL^−1 ^DNA/[PCR amplicon + plasmid length×660])×6.022×10^23^. The plasmid stock solution was diluted in nuclease-free water to a working concentration of 5×10^8^ copies per µL. Serial 10-fold dilutions were used to create external calibration curves that spanned eight orders of magnitude ranging from 5×10^7^ to 5×10^−1^ copies per µL. Two µL of each dilution were used per reaction, yielding a standard curve that ranged from 1×10^1^ to 1×10^8^ copies. The standard curve for each assay was obtained by plotting the log of the calculated copy number against the cycle at which fluorescence for that sample crossed the threshold value (cycle threshold, C_T_) (Figures S1, S2, S3, and S4). The slope, y-intercept, r^2^ value, and PCR efficiency of each assay can be found in the Supporting Information (Figures S1, S2, S3, and S4).

### Quantitative PCR

The Fast SYBR Green Master Mix (Applied Biosystems, Foster City, CA) was used for all qPCR assays. Each reaction contained: 10 µL 2x Fast SYBR Green master mix, 150–300 nM each forward and reverse primer, 5 ng (2 µL) template cDNA, and brought to a final volume of 20 µL with nuclease-free water. Reactions were conducted in 96-well plates on a ViiA 7 with the Fast 96-well block system (Applied Biosystems, Foster City, CA). The following protocol was used for all assays: an initial 20 s incubation at 95°C, followed by 40 cycles of 95°C for 1 s and 60°C for 20 s, followed by a melt curve analysis of 95°C for 15 s, 60°C for 1 min, and 95°C for 15 s to determine product specificity. All qPCR reactions were performed in triplicate using Applied Biosystems MicroAmp Fast 96-well reaction plates (0.1 mL with barcode) sealed with MicroAmp optical adhesive film. No-template controls were also included in each amplification run to monitor for contamination. Reactions were recorded and analyzed using the Applied Biosystems ViiA 7 System software. The raw C_T_ values and intra-assay variations for both relative and absolute quantification assays are provided in Data S1 and Data S2.

### Data analysis

Three types of environmental conditions were used to determine the appropriateness of a suite of genes for use as reference genes: total darkness, in which samples were collected over a time span of 70 hrs; light/dark cycling, in which samples were collected from alternating periods of light and dark over the span of ca. 70 hrs; and temperature fluctuations in darkness, in which samples were collected from alternating cycles of 25°C and 37°C over the span of ca. 70 hrs. Expression levels of all twelve candidate reference genes were initially determined from their C_T_ values. The expression stability of all candidate reference genes under each of the different conditions and among all conditions combined was assessed using the BestKeeper v. 1 and Normfinder software programs. For data input into NormFinder, C_T_s of each gene at each time point were converted to relative quantities using the equation Q = *E* ^ΔC^_T_, where ΔC_T_ = C_T_ highest abundant sample – C_T_ sample, *E* = PCR efficiency, and Q = relative quantity.

The number of transcripts and gene copies per mL were calculated via external calibration curves for *act*, *adk*, *asl*, and *vma3*. Transcript or gene copy numbers from total RNA or DNA extracted from 1 mL bioreactor samples were calculated automatically from the regression lines by the ViiA 7 System software. Gene copies were calculated using an equation described previously [19], in which gene copies per mL sample = (gene copies per reaction mix x volume of DNA [µL])/(DNA per reaction mix [µL]×mL sample used) and adjusted to account for the 1∶5 initial dilution factor. The following equation was used to calculate transcript copies per mL: [(copies per µL per reaction mix×RNA dilution factor)/(volume µL total RNA added to RT reaction/RT reaction volume µL×cDNA dilution factor]×vol total RNA eluted (µL) (Data S2 and Data S3). The transcript to gene ratio was determined by dividing transcript copies per mL by gene copies per mL. The Shapiro-Wilkes test was used to test for normality of both the ratio and C_T_ values (Table S2). If both data sets passed the test for normality, the Pearson product moment correlation test was applied to examine the strength of the correlation between the two. If one of the data sets failed the test for normality, the Spearman rank order correlation test was applied.

## Results and Discussion

### Quantitative PCR assay design, optimization, and general characteristics

Typical reference genes include GAPDH, albumin, actins, tubulins, and small and large subunit rRNA [5], as they are present in all nucleated cell types and are necessary for basic cell survival. However, numerous studies, across different organisms, have shown that the typical reference genes are regulated and vary under different conditions [5]. Based on the experimental evidence thus far, it appears that no reference gene retains sufficient overall expression stability suitable for all contexts and that the best candidates differ between tissue type [20] and environmental condition. Therefore, it is recommended that the individual investigator choose a reference gene that is most stably expressed under their specific conditions, such as tissue type or stage of development, and should be impervious to perturbations such as external stimuli, disease, or genetic modification [5], [20]–[22].

Several approaches exist for qPCR data normalization. One approach recently implemented has been to select genes from a genome-wide background derived from large sets of microarray data. This approach has been used for *Arabadopsis*, multiple human cell lines and cancers, and mouse [20]. However, while *N. crassa* serves as a model organism for studying eukaryotic circadian pathways, it is not included in this database. Additionally, studies utilizing whole-transcriptome profiling such as microarrays and RNA sequencing are able to select appropriate reference genes based on the transcriptomic profiling. For example, genes encoding an adenosine kinase and the ATP synthase gamma chain have been used as reference genes in the filamentous fungus *Penicillium glabrum* under conditions of heat stress, as their expression was shown via initial microarray analysis to be unaffected by this condition [23]. Additionally, a recent study evaluated 12 potential reference genes in the yeast *S. cerevisiae* based on pooled data from publicly-available transcriptome studies. The majority of selected genes were not previously reported as reference genes and the results revealed that these newly-identified genes outperformed the commonly-used reference genes [24]. There is extremely limited gene expression data with *N. crassa* growth in bioreactors, such that large data sets from which to select potential housekeeping genes do not exist. Also lacking are publicly-available whole-transcriptome data sets such as has been used for other organisms when selecting reference genes. Therefore, potential reference genes were selected based on the following features: previous transcriptomic-based studies from *N. crassa* (*acl*) [25] or other fungi (*adk*, *asl*) [23] in which the expression of these genes remained unchanged; via selection from the Broad Institute *N. crassa* database (http://www.broadinstitute.org/annotation/genome/neurospora/MultiHome.html) with genes for which no expression data existed (*tbp*), or from previous studies of genes whose results pertaining to their function and regulation indicated the potential for use as a reference gene (*vma1*, *vma2*, *vma3*, trk) [26], [27] Additionally, *N. crassa* genes employed as reference genes in previous studies were also evaluated here (*act*, *btl*, *l6*, *frh*) [28], albeit with newly-designed primers for use in the *btl* and *frh* assays.

**Figure 3:**
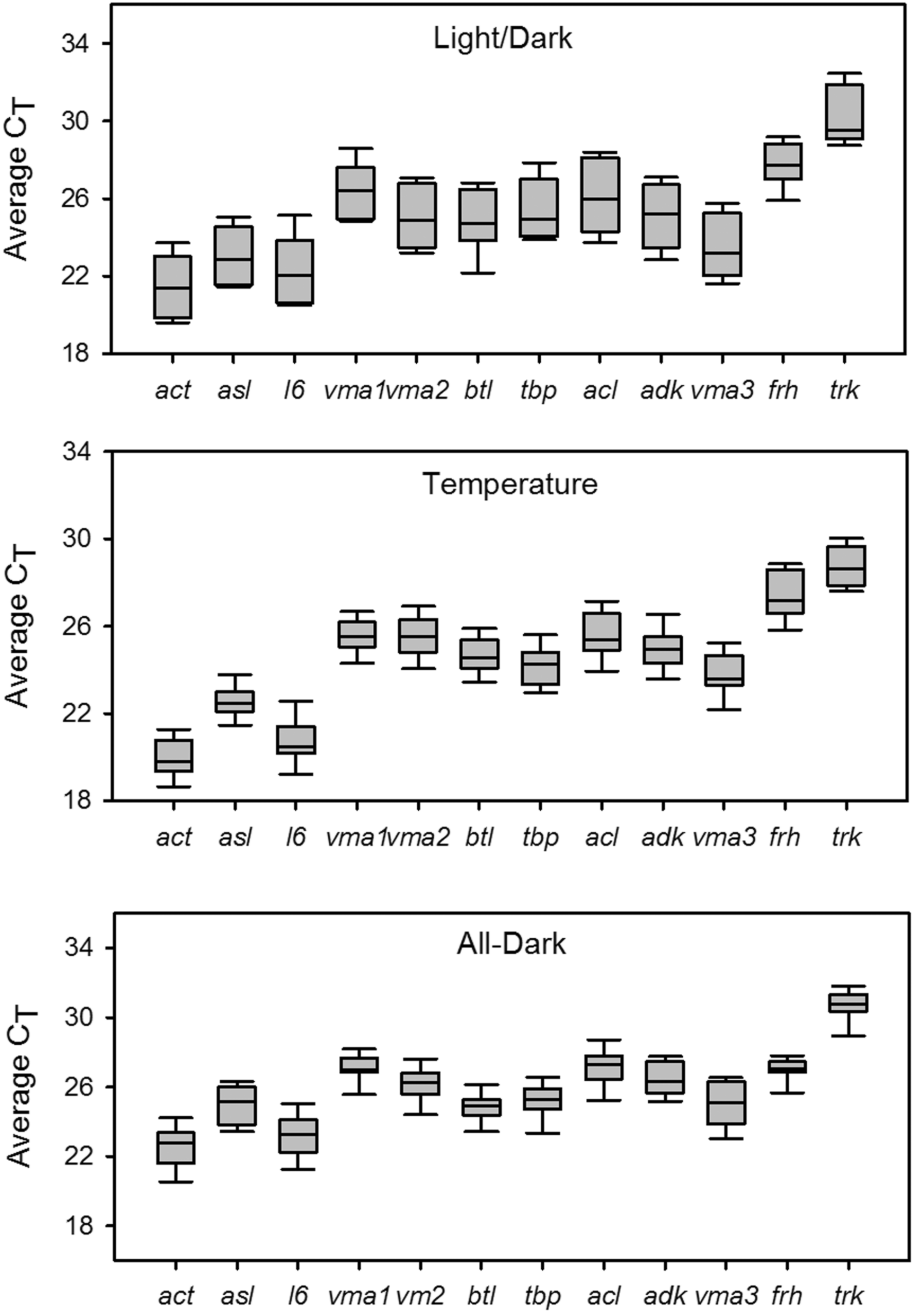
Boxplot of C_T_ values of candidate reference genes during light/dark cycling, temperature fluctuation, and continuous darkness. A line across the box depicts the median. The box indicates the 25th and 75th percentiles. Whiskers represent the 90th and 10th percentiles.

The compliance of the RT-qPCR assays with the MIQE (Minimum Information for Publication of Quantitative Real-Time PCR Experiments [29]) guidelines is shown in the MIQE checklist (Table S3). All assays developed in this study were specific for their intended gene target, as indicated by a single peak in the melt curve analysis (Figure S5). Additionally, all possessed similar PCR efficiencies (Table 1), so that if all had displayed equal stability, they could have been used interchangeably in relative quantification methods. A preliminary assessment of the 12 reference genes showed that average C_T_ values, when assimilating the data from all genes, ranged from 21.47–30.34 under light/dark fluctuations (Figure 3A), 22.53–30.72 under all dark conditions (Figure 3B), and 19.93–28.73 under temperature flux (Figure 3C). Based on this initial analysis, the two genes displaying the highest average C_T_ values, *frh* and *trk*, were discarded from further analysis. This was due to the recommendation of reference genes being expressed at comparable levels to both other reference genes and target genes [30]. Additionally, *frh* has been shown to be under clock control [31].

### Reference gene stability under light/dark conditions

The expression stability of candidate reference genes was initially estimated based on calculated variations (SD, standard deviation of the C_T_, and CV, the coefficient of variance expressed as a percentage on the C_T_ level) using BestKeeper. With this program, reference genes can be ordered from the most stably expressed, exhibiting the lowest variation, to the least stable. In addition to stability (SD and CV) values, additional key parameters include the Pearson correlation coefficient (*r*), in which pair-wise correlation analyses are performed to estimate inter-gene relationships among all reference genes, and their associated probability (*p*) values. Thus, *r* and *p* describe the relation between the BestKeeper index and each gene. The candidate reference genes were then ranked as to their suitability under one environmental condition and across all three using the program NormFinder. This program utilizes a model-based approach that, in addition to assessing overall expression variation, allows for the samples to be assigned to different groups, allowing for the intra- and intergroup expression variation of the candidate genes to be assessed; this grouping feature was used when assessing reference gene stability under light/dark and temperature cycling. It was not employed for all-dark growth at constant temperature. The comprehensive results of the BestKeeper and NormFinder programs are provided in Data S4 and Data S5, respectively.

The greatest variability among the three environmental conditions was observed under light/dark fluctuation, as evidenced by both the range in C_T_s and the SD values obtained with BestKeeper (Table 2). In general, most genes displayed overall stability with respect to light/dark cycling. Three genes (*btl*, *asl*, and *vma1*) displayed the greatest expression stability (SD = 1.10, 1.13, and 1.21, respectively), while *l6* and *acl* were the least stable (SD = 1.41). However, based on the Pearson correlation coefficients (*r*), they correlated well with the other genes and thus were retained for further analysis per previous methods [12]. Overall, genes displayed similar Pearson correlation coefficients, ranging between 0.936–0.997. NormFinder identified *vma1* as the most stable gene (Table 2), with the best combination of two genes being *vma1* and *asl*. The similarity in stability values between *vma1* and *asl* was also reflected in the intragroup variation, which was 0.001 in the light for both. However, *vma1* expression was less influenced under dark conditions than *asl*, with an intragroup variation of 0.002 versus the 0.016 calculated for *asl* (Table 2).

**Table 2:** Descriptive statistics and stability values of candidate reference genes under conditions of light/dark cycling (n = 8).

	*btl*	*asl*	*vma1*	*act*	*vma2*	*tbp*	*adk*	*vma3*	*acl*	*l6*
**geo Mean [C_T_]**	24.60	22.72	26.24	21.15	24.82	25.21	24.77	23.33	25.85	22.00
**ar Mean [C_T_]**	24.63	22.75	26.28	21.19	24.86	25.25	24.81	23.38	25.90	22.06
**min [C_T_]**	22.16	21.44	24.85	19.60	23.21	23.88	22.84	21.62	23.73	20.51
**max [C_T_]**	26.76	24.91	28.59	23.47	27.05	27.86	26.89	25.75	28.17	25.14
**SD [±C_T_]**	**1.10**	**1.13**	**1.21**	**1.24**	**1.24**	**1.26**	**1.34**	**1.38**	**1.41**	**1.41**
**CV [% C_T_]**	4.482	4.967	4.608	5.834	4.990	5.004	5.381	5.881	5.449	6.397
***r***	0.936	0.997	0.997	0.988	0.995	0.987	0.970	0.985	0.954	0.985
***p***	0.001	0.001	0.001	0.001	0.001	0.001	0.001	0.001	0.001	0.001
**NF Stability value**	**0.194**	**0.070**	**0.067**	**0.154**	**0.114**	**0.169**	**0.175**	**0.252**	**0.278**	**0.274**
**Int Var - Light**	0.062	0.001	0.001	0.033	0.001	0.157	0.140	0.001	0.134	0.342
**Int Var - dark**	0.105	0.016	0.002	0.039	0.002	0.008	0.032	0.026	0.145	0.028

NF = NormFinder, Int Var = Intragroup Variation as calculated by Normfinder.

### Reference gene stability under all-dark conditions

Based on the SD as calculated by BestKeeper, all genes were stable during complete darkness, as the SD for all genes was <1 (Table 3). The only exception to this was *vma3*, in which the SD = 1.07; however, the Pearson correlation coefficient was acceptable, and in fact higher than some of the other genes that displayed a lower SD than *vma3*. Genes displaying the lowest SD, and thus the greatest stability, were *vma1*, *btl*, and *tbp*. All genes were significantly correlated to the BestKeeper index (p<0.001), and the Pearson correlation coefficient was very similar among genes. In general, the analysis showed a strong correlation for all 10 genes. NormFinder identified *tbp*, *vma2*, and *vma1* as being the most stable of the genes under conditions of complete darkness, with *tbp* and *vma2* recording almost identical stability values (0.141 and 0.143, respectively) (Table 3).

**Table 3:** Descriptive statistics and stability values of candidate reference genes under all-dark conditions (n = 10).

	*vma1*	*btl*	*tbp*	*vma2*	*acl*	*adk*	*asl*	*act*	*l6*	*vma3*
**geo Mean [C_T_]**	27.07	24.84	25.22	26.15	27.16	26.46	24.96	22.50	23.18	24.90
**ar Mean [C_T_]**	27.08	24.85	25.24	26.17	27.17	26.48	24.98	22.53	23.21	24.93
**min [C_T_]**	25.44	23.32	23.20	24.27	25.11	25.15	23.40	20.43	21.14	22.97
**max [C_T_]**	28.22	26.19	26.58	27.63	28.79	27.78	26.33	24.29	25.09	26.55
**SD [±C_T_]**	**0.489**	**0.584**	**0.699**	**0.729**	**0.739**	**0.777**	**0.899**	**0.932**	**0.989**	**1.074**
**CV [% CP]**	1.807	2.349	2.768	2.787	2.718	2.936	3.600	4.134	4.260	4.308
***r***	0.931	0.886	0.98	0.986	0.943	0.734	0.934	0.983	0.990	0.971
***p***	0.001	0.001	0.001	0.001	0.001	0.016	0.001	0.001	0.001	0.001
**NF Stability Value**	**0.169**	**0.181**	**0.141**	**0.144**	**0.211**	**0.499**	**0.398**	**0.268**	**0.271**	**0.261**

NF = NormFinder.

### Reference gene stability during temperature fluctuation

All genes were stably expressed under conditions of temperature fluctuations, as reflected by the SD ranging from 0.501–0.865. Overall, SDs ranged between 0.501–0.741, with the exceptions of *l6* (0.796) and *acl* (0.865). Based on the SD, *asl* (0.501), *btl* (0.616), and *vma1* (0.622) exhibited the greatest stability (Table 4). As had been observed for all-dark conditions, all genes correlated very strongly with the index, with most displaying *r*>0.9. Under conditions of fluctuating temperature, Normfinder identified *vma1* as the most stable, with the best combination being *vma1* and *act* (Table 4). *vma1* displayed extremely low variation under 25°C (0.0001) with an increase in variance observed at 37°C for both *vma1* and *act* (0.026 and 0.018, respectively).

**Table 4:** Descriptive statistics and stability values of candidate reference genes under temperature fluctuations (n = 12).

	*asl*	*btl*	*vma1*	*adk*	*act*	*tbp*	*vma2*	*vma3*	*l6*	*acl*
geo Mean [C_T_]	22.51	24.60	25.55	24.93	19.91	24.19	25.52	23.74	20.70	25.55
ar Mean [C_T_]	22.52	24.62	25.56	24.95	19.93	24.20	25.54	23.76	20.72	25.57
min [C_T_]	21.37	23.31	24.27	23.49	18.60	22.94	23.83	22.03	19.04	23.85
max [C_T_]	23.87	26.01	26.69	26.66	21.38	25.68	27.10	25.37	22.64	27.36
**SD [±C_T_]**	**0.501**	**0.616**	**0.622**	**0.673**	**0.675**	**0.693**	**0.718**	**0.741**	**0.796**	**0.865**
CV [% C_T_]	2.225	2.504	2.435	2.698	3.387	2.863	2.811	3.117	3.841	3.381
*r*	0.953	0.865	0.970	0.792	0.978	0.906	0.837	0.998	0.944	0.950
*p*	0.001	0.001	0.001	0.002	0.001	0.001	0.001	0.001	0.001	0.001
**NF Stability value**	**0.133**	**0.175**	**0.073**	**0.262**	**0.087**	**0.133**	**0.175**	**0.090**	**0.165**	**0.190**
Int Var 25°C	0.022	0.074	0.000	0.012	0.015	0.006	0.115	0.011	0.153	0.066
Int Var 37°C	0.006	0.041	0.026	0.266	0.018	0.093	0.017	0.003	0.050	0.271

NF = NormFinder, Int Var = Intragroup Variation as calculated by Normfinder.

### Reference gene stability across all conditions

Assimilating the results of both BestKeeper and NormFinder under each individual condition, *vma1* always ranked among the top three genes in terms of stability; *asl* and *btl* also consistently ranked in the top three in at least one of the analyses. Also, in keeping with their biological role as subunits of the same enzyme, *vma1*, *vma2*, and, under several conditions, *vma3* ranked closely together in terms of stability, suggesting they can be used interchangeably. However, in order to accurately identify the most stable genes under all three conditions, all data were combined and analyzed with both BestKeeper and Normfinder. The results of BestKeeper revealed that 4 of the 10 genes displayed acceptable stability across all conditions (reflected by a SD<1): *btl*, *tbp*, *vma2*, and *vma1* (Table 5). Conversely, the two genes frequently used for normalization, *l6* and *act*, displayed the greatest variance (Table 5). The most appropriate gene for use across all conditions as identified via NormFinder was *vma1*, with the best combination consisting of *vma1* and *asl*, with a combined stability value of 0.089. *tbp* and *vma2* were the next most-stable genes among the 10, in agreement with the results obtained with BestKeeper (Table 5).

**Table 5:** Descriptive statistics and stability values of candidate reference genes under light/dark cycling, all-dark growth, and temperature fluctuations (n = 30).

	*btl*	*tbp*	*vma2*	*vma1*	*adk*	*vma3*	*acl*	*asl*	*act*	*l6*
geo Mean [C_T_]	24.68	24.80	25.54	26.23	25.39	24.01	26.16	23.35	21.08	21.85
ar Mean [C_T_]	24.70	24.83	25.57	26.26	25.42	24.05	26.19	23.40	21.13	21.91
min [C_T_]	22.16	22.94	23.21	24.27	22.84	21.62	23.73	21.37	18.60	19.04
max [C_T_]	26.76	27.86	27.63	28.59	27.78	26.55	28.79	26.33	24.29	25.14
**SD [±C_T_]**	**0.750**	**0.927**	**0.956**	**0.982**	**1.088**	**1.137**	**1.193**	**1.257**	**1.341**	**1.415**
CV [% C_T_]	3.036	3.733	3.739	3.741	4.280	4.729	4.555	5.370	6.344	6.459
*r*	0.810	0.903	0.880	0.973	0.892	0.951	0.961	0.939	0.955	0.962
*p*	0.001	0.001	0.001	0.001	0.001	0.001	0.001	0.001	0.001	0.001
**NF Stability value**	**0.191**	**0.137**	**0.178**	**0.129**	**0.230**	**0.243**	**0.279**	**0.148**	**0.185**	**0.218**
Int Var: Dark Flux	0.129	0.003	0.007	0.004	0.022	0.019	0.129	0.023	0.040	0.025
Int Var: Light Flux	0.089	0.136	0.001	0.001	0.150	0.001	0.166	0.002	0.025	0.300
Int Var: All Dark	0.040	0.024	0.020	0.032	0.254	0.058	0.051	0.156	0.066	0.067
Int Var: 25°C	0.074	0.006	0.115	0.000	0.012	0.011	0.066	0.022	0.015	0.153
Int Var: 37°C	0.041	0.093	0.017	0.026	0.266	0.003	0.271	0.006	0.018	0.050

NF = NormFinder, Int Var = Intragroup Variation as calculated by Normfinder.

### Absolute quantification of transcript and gene copy numbers

**Figure 4:**
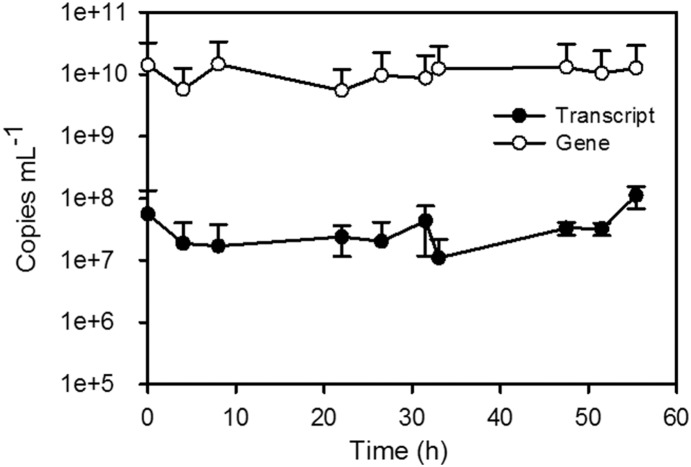
Representative figure of data used to obtain transcript-to-gene ratios. *act* all-dark transcript and gene copies per mL of sample were calculated via absolute quantification using external calibration curves. Error bars represent the standard deviation derived from triplicate qPCRs from biological replicates.

In bioreactors, *N. crassa* exists as different vegetative cell types, some of which are multi-nucleated. Based on the parameters [6], [12] defined for appropriate reference gene selection, all 10 candidate reference genes displayed fluctuations in expression. This prompted the question: Were fluctuating C_T_s due to changes in gene expression, or were gene (DNA) copy numbers changing, so that if transcript levels remained relatively stable, it was manifested as expression fluctuations? Therefore, expression changes in a subset of the candidate genes was assessed using absolute quantification. The transcript (RNA) and gene (DNA) copy numbers of *act*, *adk*, *asl*, and *vma3*, were calculated and used as an additional means of examining gene expression. These four were selected based on combination of factors since they are involved in different biological processes within the cell and ranged in their stability values under the conditions examined. For example, *asl* frequently ranked as one of the most stable, while *adk* was one of the least stable. External standards were created and used to calculate both transcript and gene copy numbers per mL of sample in order to determine the ratio of transcript copies to gene copies over time (representative image in Figure 4, plots of transcript and gene copy numbers for all genes under all conditions Figures S6, S7, S8, and S9). This ratio indicated whether C_T_ fluctuations were due to changes in transcript abundance, indicative of changes in gene expression, or whether it was a result of changes in gene copy number, such as could occur in multinucleated cells.

**Figure 5:**
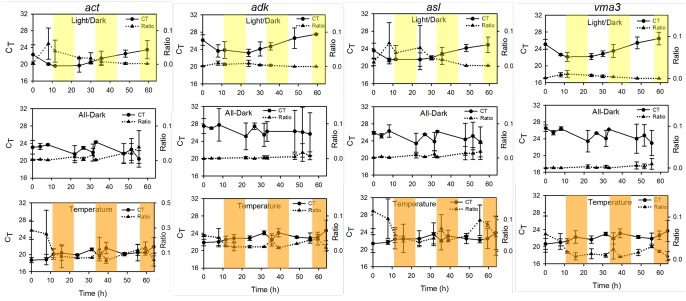
Correlation between gene expression and C_T_ values as derived from absolute quantification of transcript and gene copy numbers. Standard curves were created for the *act*, *adk*, *asl*, and *vma3* genes and used to calculate the transcript-to-gene ratios under conditions of light/dark cycling, temperature fluctuation, and continuous darkness. Top panel depicts light/dark cycling, with yellow highlight indicating light on. Bottom panel displays conditions of temperature flux between 25°C and 37°C, with 37°C illustrated by orange highlight.

Plotting both the RNA: DNA ratio and C_T_ values over time indicate that C_T_ changes are due to fluctuations in transcript copy number (Figure 5). The Pearson Product Correlation test was applied to the ratio and corresponding C_T_ of each time point for each gene to test the hypothesis that a negative correlation exists between ratio and C_T_ (i.e. as the ratio increases, the C_T_ decreases). With all four genes under all three conditions, a strong negative correlation was found to exist between ratio and C_T_ values, demonstrating that C_T_ changes serve as a reliable reflection of transcript, and not gene copy number fluctuations (Table 6). For example, under conditions of light/dark cycling, the *act* C_T_ value decreased at the second time point (Figure 5A), indicative of an increase in transcripts. If this were due to changes in gene (DNA) copy number, the RNA: DNA ratio would not increase. However, an increase in ratio indicates an increase in transcript abundance. As statistically significant negative correlations were found to exist between the transcript: gene ratio and C_T_ values among the four genes under all conditions, these data demonstrate that even with the presence of different cell types, relative quantification is an acceptable method for measuring gene expression changes in *N. crassa* during growth in bioreactors.

**Table 6:** Pearson Product Correlation values obtained for ratio and CT of *act*, *adk*, *asl*, and *vma3* under each condition.

Gene/Condition	*r*	p-value
*act* Light/Dark	−0.747	0.033
*act* All Dark	−0.868	0.001
*act* Temperature	−0.685*	0.012
*asl* Light/Dark	−0.880	0.004
*asl* All Dark	−0.765	0.010
*asl* Temperature	−0.721	0.008
*adk* Light/Dark	−0.924	0.001
*adk* All Dark	−0.636*	0.040
*adk*Temperature	−0.721	0.008
*vma3* Light/Dark	−0.921	0.001
*vma3* All Dark	−0.939*	0.000
*vma3* Temperature	−0.748	0.004

*indicates Spearman rank value.

## Conclusions

The work presented here mirrors the results obtained in recent studies with multiple organisms in which the most appropriate reference genes for use in RT-qPCR were not those commonly employed when using the relative quantification method, underscoring the importance of proper reference gene evaluation and selection. The increased use of RT-qPCR coupled with the realization of the importance of selecting the appropriate reference gene has led to dedicated studies on reference gene selection in both model and non-model organisms, including rats [32], [33], zooplankton [34], fruit crops [35] (*Litchi chinensis*), and insects [36]–[39]. In some cases, no single gene was found to be stable under all conditions, with stability affected by both endogenous and exogenous factors such as cell type, developmental stage, diet, and environmental stressors [38], [39]. Additionally, reference gene studies have been undertaken in recent years with multiple genera of filamentous fungi. A study evaluating potential reference genes in *Trichoderma reesei*, similar in industrial importance to *N. crassa* due to its high secretory capacity for hydrolases, identified *sar1*, encoding a small GTPase, as most stable, whereas the gene coding for actin (*act*), did not rank among the best validated ones [40]. In the filamentous fungus *Fusarium graminearum*, an economically-important plant pathogen, evaluation of 15 genes previously identified as housekeeping genes or those selected from the whole transcriptome sequencing data under different culture conditions found the most appropriate reference genes to vary based on growth stage and/or toxin production [41] and were comprised of genes coding for a cell surface flocculin, ubiquitin C-terminal hydrolase, the eukaryotic translation initiation factor 1 alpha, and the mitochondrial ribosomal protein S16.

In this study, we identified and validated new reference genes for use in *N. crassa* expression studies under multiple environmental conditions, including long-term growth in constant darkness, light/dark cycling, and temperature flux in constant darkness. In addition to different environmental conditions, we assessed the time-course variation in reference gene expression by sampling at frequent intervals over a period of >60 hours. These diverse conditions and continuous culture conditions span primary developmental (mycelia, macro- and microconidia) and/or physiological stages, thus increasing the reliability of the reference genes selected in this study. Overall, the results demonstrate that, under the culture conditions described here, *vma1*, *vma2*, *tbp*, and *btl* serve as the most appropriate reference genes for use in qPCR. We also demonstrated that *l6*, and to a lesser extent *act*, often used in the relative quantification method, are less suitable as reference genes in comparison to multiple other genes.

## Supporting Information

Figure S1 **Standard curve of** ***act*** **assay.** A plasmid-based standard was constructed for the actin gene via amplification from genomic DNA using actin-specific primers. The number of copies per µL was calculated using the equation: X g µL-1 DNA/[PCR amplicon+plasmid length×660])×6.022×1023. Serial 10-fold dilutions were used to create external calibration curves that spanned eight orders of magnitude ranging from 1×10^1^ to 1×10^8^ copies. The standard curve for each assay was obtained by plotting the log of the calculated copy number against the cycle at which fluorescence for that sample crossed the threshold value. The slope, y-intercept, r^2^ value, and PCR efficiency of the actin assay is provided in the figure.
(TIF)

Figure S2 **Standard curve of** ***adk*** **assay, constructed as described for the act assay.**
(TIF)

Figure S3 **Standard curve of** ***asl*** **assay, constructed as described for the act assay.**
(TIF)

Figure S4 **Standard curve of** ***vma3*** **assay, constructed as described for the act assay.**
(TIF)

Figure S5 **Melt curve profiles of the 12 reference gene assays evaluated in this study.**
(TIF)

Figure S6 ***act*** **transcript and gene copies over time under conditions of light/dark cycling, temperature flux, and continuous darkness.** Transcript and gene copies per mL of sample were calculated via absolute quantification using external calibration curves as described in the text. Error bars represent the standard deviation derived from triplicate qPCRs from biological replicates.
(TIF)

Figure S7 ***adk*** **transcript and gene copies over time under conditions of light/dark cycling, temperature flux, and continuous darkness.** Transcript and gene copies per mL of sample were calculated via absolute quantification using external calibration curves as described in the text. Error bars represent the standard deviation derived from triplicate qPCRs from biological replicates.
(TIF)

Figure S8 ***asl*** **transcript and gene copies over time under conditions of light/dark cycling, temperature flux, and continuous darkness.** Transcript and gene copies per mL of sample were calculated via absolute quantification using external calibration curves as described in the text. Error bars represent the standard deviation derived from triplicate qPCRs from biological replicates.
(TIF)

Figure S9 ***vma3*** **transcript and gene copies over time under conditions of light/dark cycling, temperature flux, and continuous darkness.** Transcript and gene copies per mL of sample were calculated via absolute quantification using external calibration curves as described in the text. Error bars represent the standard deviation derived from triplicate qPCRs from biological replicates.
(TIF)

Table S1 **Primers used in construction of** ***act*** **,** ***adk*** **,** ***asl*** **, and** ***vma3*** **external standards for absolute quantification.**
(PDF)

Table S2 **Normality assessment of transcript: gene ratio and C_T_ values of** ***act*** **,** ***adk*** **,** ***asl*** **, and** ***vma3*** **genes by Shapiro-Wilkes test.**
(PDF)

Table S3 **MIQE checklist.**
(XLS)

Data S1 **Raw C_T_ values and intra-assay variations for 12 reference genes assessed using relative quantification.**
(XLSX)

Data S2 **Raw C_T_ values and intra-assay variations for reference genes assessed using absolute quantification.**
(XLSX)

Data S3 **Sample description, total RNA concentration, dilution factor, and volume added to reverse transcription reaction.**
(XLSX)

Data S4 **Comprehensive output of BestKeeper analysis.**
(XLSX)

Data S5 **Comprehensive output of NormFinder analysis.**
(XLSX)

## References

[1] Baker CL, Loros JJ, Dunlap JC (2012) The circadian clock of *Neurospora crassa* . FEMS Microbiology Reviews 36:95–110. 21707668 10.1111/j.1574-6976.2011.00288.x PMC3203324

[2] Jacobson DJ, Powell AJ, Dettman JR, Saenz GS, Barton MM, et al (2004) *Neurospora* in temperate forests of western North America. Mycologia 96:66–74. 21148830

[3] Dogaris I, Mamma D, Kekos D (2013) Biotechnological production of ethanol from renewable resources by *Neurospora crassa:* an alternative to conventional yeast fermentations? Appl Microbiol Biotechnol 97:1457–1473. 23318834 10.1007/s00253-012-4655-2

[4] Glass NL, Schmoll M, Cate JHD, Coradetti S (2013) Plant cell wall deconstruction by ascomycete fungi. In: Gottesman S, editor. Annual Review of Microbiology, Vol 67. Palo Alto: Annual Reviews. pp. 477–498. 10.1146/annurev-micro-092611-150044 23808333

[5] Pfaffl MW (2004) Quantification strategies in real-time PCR. In: Bustin SA, editor. A–Z of quantitative PCR. La Jolla, CA, USA: International University Line. pp. 87–112.

[6] Vandesompele J, De Preter K, Pattyn F, Poppe B, Van Roy N, et al (2002) Accurate normalization of real-time quantitative RT-PCR data by geometric averaging of multiple internal control genes. Genome Biology 3:research0034.0031–0034.0011. 12184808 10.1186/gb-2002-3-7-research0034 PMC126239

[7] Bustin SA, Nolan T (2004) Pitfalls of quantitative real-time reverse-transcription polymerase chain reaction. J Biomol Tech 15:155–166. 15331581 PMC2291693

[8] Bistis GN, Perkins DD, Read ND (2003) Different cell types in *Neurospora crassa* . Fungal Genet Newsl 50:17–19.

[9] Sun J, Tian C, Diamond S, Glass NL (2012) Deciphering transcriptional regulatory mechanisms associated with hemicellulose degradation in *Neurospora crassa* . Euk Cell 11:482–493. 10.1128/EC.05327-11 PMC3318299 22345350

[10] Coradetti ST, Craig JP, Xiong Y, Shock T, Tian C, et al (2012) Conserved and essential transcription factors for cellulase gene expression in ascomycete fungi. Proc Natl Acad Sci USA 109:7397–7402. 22532664 10.1073/pnas.1200785109 PMC3358856

[11] Tralau T, Lanthaler K, Robson GD, Crosthwaite SK (2007) Circadian rhythmicity during prolonged chemostat cultivation of *Neurospora crassa* . Fungal Genetics and Biology 44:754–763. 17196855 10.1016/j.fgb.2006.11.003

[12] Pfaffl MW, Tichopad A, Prgomet C, Neuvians TP (2004) Determination of stable housekeeping genes, differentially regulated target genes and sample integrity: BestKeeper – Excel-based tool using pair-wise correlations. Biotechnol Lett 26:509–515. 15127793 10.1023/b:bile.0000019559.84305.47

[13] Andersen CL, Ledet-Jensen J, Orntoft T (2004) Normalization of real-time quantitative RT-PCR data: a mode-based variance estimation approach to identify genes suited for normalization, applied to bladder and colon cancer data sets. Cancer Res 64:5245–5250. 15289330 10.1158/0008-5472.CAN-04-0496

[14] Vogel HL (1956) A conveniant medium for *Neurospora* (medium N). Microbial Genet Bull 13:42–43.

[15] Morrison TB, Weiss JJ, Wittwer CT (1998) Quantification of low-copy number transcripts by continuous SYBR Green I monitoring during amplification. Biotechniques 24: 954–958, 960, 962. 9631186

[16] Zuker M (2003) Mfold web server for nucleic acid folding and hybridization prediction. Nucleic Acids Res 31:3406–3415. 12824337 10.1093/nar/gkg595 PMC169194

[17] Ramakers C, Ruijter JM, Deprez RHL, Moorman AFM (2003) Assumption-free analysis of quantitative real-time polymerase chain reaction (PCR) data. Neuroscience Letters 339:62–66. 12618301 10.1016/s0304-3940(02)01423-4

[18] Pfaffl MW, Hageleit M (2001) Validities of mRNA quantification using recombinant RNA and recombinant DNA external calibration surves in real-time RT-PCR. Biotech Lett 2#: 275–282.

[19] Ritalahti KM, Amos BK, Sung Y, Wu S, Koenigsberg SS, et al (2006) Quantitative PCR targeting 16S rRNA and reductive dehalogenase genes simulateously monitors multiple *Dehalococcoides* strains. Appl Environ Microbiol 72:2765–2774. 16597981 10.1128/AEM.72.4.2765-2774.2006 PMC1449079

[20] Hruz T, Wyss M, Docquier M, Pfaffl M, Masanetz S, et al (2011) RefGenes: identification of reliable and condition specific reference genes for RT-qPCR data normalization. BMC Genomics 12:156. 21418615 10.1186/1471-2164-12-156 PMC3072958

[21] Huggett J, Dheda K, Bustin S, Zumla A (2005) Real-time RT-PCR normalization: strategies and considerations. Genes and Immunity 6:279–284. 15815687 10.1038/sj.gene.6364190

[22] Gutierrez L, Mauriat M, Pelloux J, Bellini C, Wuytswinkel OV (2008) Towards a systematic validation of references in real-time RT-PCR. The Plant Cell 20:1734–1735. 18664615 10.1105/tpc.108.059774 PMC2518241

[23] Nevarez L, Vasseur V, Le Drean G, Tanguy A, Guisle-Marsollier I, et al (2008) Isolation and analysis of differentially expressed genes in *Penicillium glabrum* subjected to thermal stress. Microbiology 154:3752–3765. 19047743 10.1099/mic.0.2008/021386-0

[24] Cankorur-Cetinkaya A, Dereli E, Eraslan S, Karabekmez E, Dikicioglu D, et al (2012) A novel strategy for selection and validation of reference genes in dynamic multidimensional experimental design in yeast. Plos One 7:14. 10.1371/journal.pone.0038351 PMC3366934 22675547

[25] Wang Z, Kin K, Lopez-Giraldez F, Johannesson H, Townsend JP (2012) Sex-specific gene expression during asexual development of *Neurospora crassa* . Fungal Genet Biol 49:533–543. 22626843 10.1016/j.fgb.2012.05.004 PMC3397379

[26] Wechser MA, Bowman BJ (1995) Regulation of the expression of three housekeeping genes encoding subunits of the *Neurospora crassa* vacuolar ATPase. Mol Gen Genet 249:317–327. 7500957 10.1007/BF00290533

[27] Rivetta A, Allen KE, Slayman CW, Slayman CL (2013) Coordination of K+ transporters in *Neurospora*: TRK1 Is scarce and constitutive, while HAK1 Is abundant and highly regulated. Euk Cell 12:684–696. 10.1128/EC.00017-13 PMC3647778 23475706

[28] Ish-Shalom S, Lichter A (2010) Analysis of fungal gene expression by real time quantitative PCR. In: Sharon A, editor. Methods in Molecular Biology: Molecular and Cell Biology Methods for Fungi. Clifton, NJ: Humana Press. 10.1007/978-1-60761-611-5_7 20238263

[29] Bustin SA, Benes V, Garson JA, Hellemans J, Huggett J, et al (2009) The MIQE Guidelines: Minimum Information for Publication of Quantitative Real-Time PCR Experiments. Clin Chem 55:611–622. 19246619 10.1373/clinchem.2008.112797

[30] Bustin SA (2002) Absolute quantification of mRNA using real-time reverse transcription polymerase chain reaction assays. J Mol Endocrinol 25:169–193. 10.1677/jme.0.0250169 11013345

[31] Dong W, Tang X, Yu Y, Nilsen R, Kim R, et al (2008) Systems biology of the clock in *Neurospora crassa* . PLoS One 3:e3105. 18769678 10.1371/journal.pone.0003105 PMC2518617

[32] Langnaese K, John R, Schweizer H, Ebmeyer U, Keilhoff G (2008) Selection of reference genes for quantitative real-time PCR in a rat asphyxial cardiac arrest model. BMC Mol Biol 9:15. 18505597 10.1186/1471-2199-9-53 PMC2430208

[33] Lardizabal MN, Nocito AL, Daniele SM, Ornella LA, Palatnik JF, et al (2012) Reference genes for real-time PCR quantification of microRNAs and messenger RNAs in rat models of hepatotoxicity. PLoS One 7:14. 10.1371/journal.pone.0036323 PMC3341372 22563491

[34] Spanier KI, Leese F, Mayer C, Colbourne JK, Gilbert D, et al (2010) Predator-induced defences in *Daphnia pulex*: Selection and evaluation of internal reference genes for gene expression studies with real-time PCR. BMC Mol Biol 11:11. 20587017 10.1186/1471-2199-11-50 PMC3148505

[35] Zhong HY, Chen JW, Li CQ, Chen L, Wu JY, et al (2011) Selection of reliable reference genes for expression studies by reverse transcription quantitative real-time PCR in litchi under different experimental conditions. Plant Cell Reports 30:641–653. 21301853 10.1007/s00299-010-0992-8

[36] Bansal R, Mamidala P, Mian MAR, Mittapalli O, Michel AP (2012) Validation of reference genes for gene expression studies in *Aphis glycines* (Hemiptera: Aphididae). J Econ Entomol 105:1432–1438. 22928326 10.1603/EC12095 PMC7110211

[37] Shen GM, Huang Y, Jiang XZ, Dou W, Wang JJ (2013) Effect of beta-cypermethrin exposure on the stability of nine housekeeping genes in *Bactrocera dorsalis* (Diptera: Tephritdae). Fla Entomol 96:442–450.

[38] Shen GM, Jiang HB, Wang XN, Wang JJ (2010) Evaluation of endogenous references for gene expression profiling in different tissues of the oriental fruit fly *Bactrocera dorsalis* (Diptera: Tephritidae). BMC Mol Biol 11:9. 20923571 10.1186/1471-2199-11-76 PMC2972281

[39] Shen GM, Wang XN, Dou W, Wang JJ (2012) Biochemical and molecular characterisation of acetylcholinesterase in four field populations of *Bactrocera dorsalis* (Hendel) (Diptera: Tephritidae). Pest Manag Sci 68:1553–1563. 23007913 10.1002/ps.3340

[40] Steiger MG, Mach RL, Mach-Aigner AR (2010) An accurate normalization strategy for RT-qPCR in *Hypocrea jecorina* (*Trichoderma reesei*). J Biotechnol 145:30–37. 19861137 10.1016/j.jbiotec.2009.10.012

[41] Kim HK, Yun SH (2011) Evaluation of potential reference genes for quantitative RT-PCR analysis in *Fusarium graminearum* under different culture conditions. Plant Pathology J 27:301–309.

