# Supplementary figures and images for: Selection and Evaluation of Reference Genes for Expression Studies with Quantitative PCR in the Model Fungus Neurospora crassa under Different Environmental Conditions in Continuous Culture
Source: PLoS One. 2014 Dec 4;9(12):e112706. doi: 10.1371/journal.pone.0112706 (PMC4256298; doi:10.1371/journal.pone.0112706)

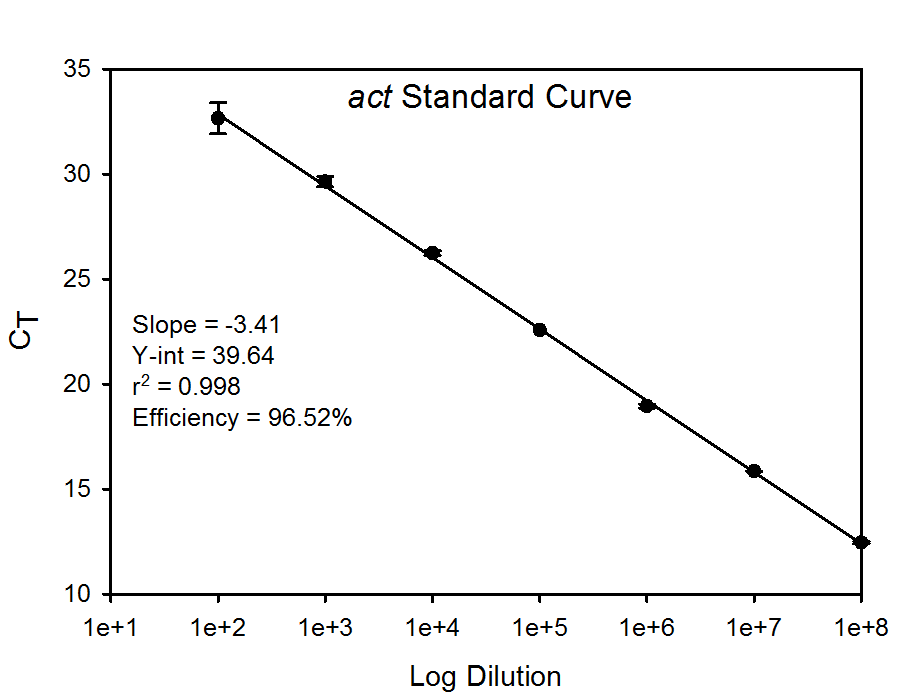

Supplement: Figure S1 — Standard curve of act assay. A plasmid-based standard was constructed for the actin gene via amplification from genomic DNA using actin-specific primers. The number of copies per µL was calculated using the equation: X g µL-1 DNA/[PCR amplicon+plasmid length×660])×6.022×1023. Serial 10-fold dilutions were used to create external calibration curves that spanned eight orders of magnitude ranging from 1×101 to 1×10^8^ copies. The standard curve for each assay was obtained by plotting the log of the calculated copy number against the cycle at which fluorescence for that sample crossed the threshold value. The slope, y-intercept, r2 value, and PCR efficiency of the actin assay is provided in the figure. (TIF) [file pone.0112706.s001.tif]

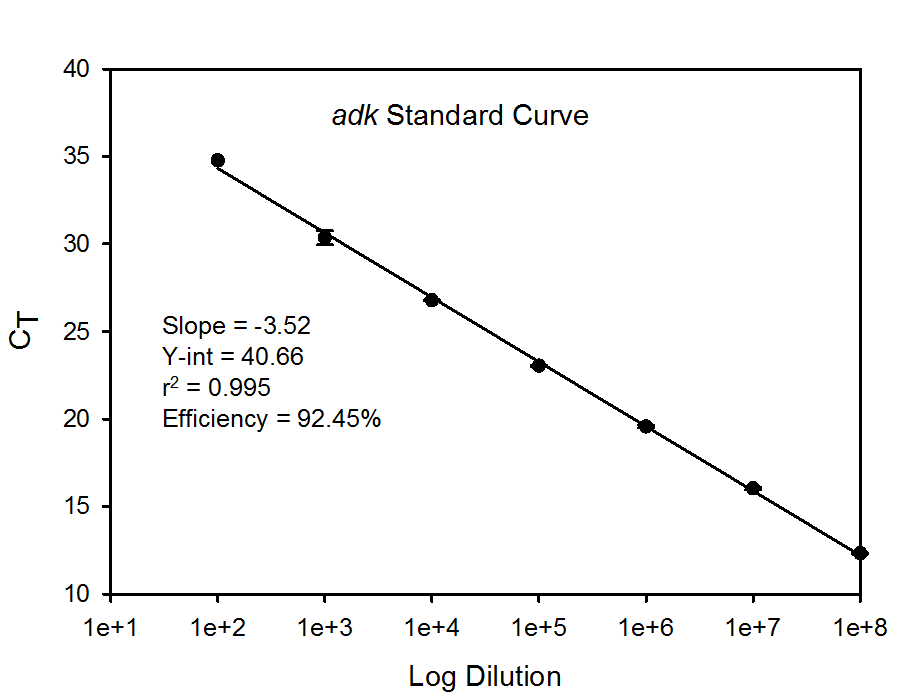

Supplement: Figure S2 — Standard curve of adk assay, constructed as described for the act assay. (TIF) [file pone.0112706.s002.tif]

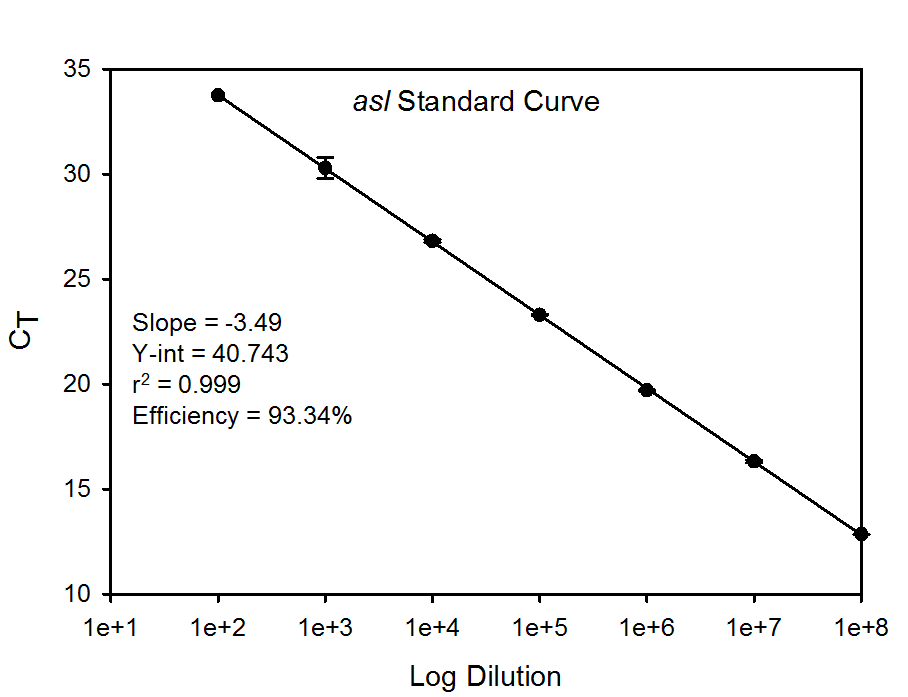

Supplement: Figure S3 — Standard curve of asl assay, constructed as described for the act assay. (TIF) [file pone.0112706.s003.tif]

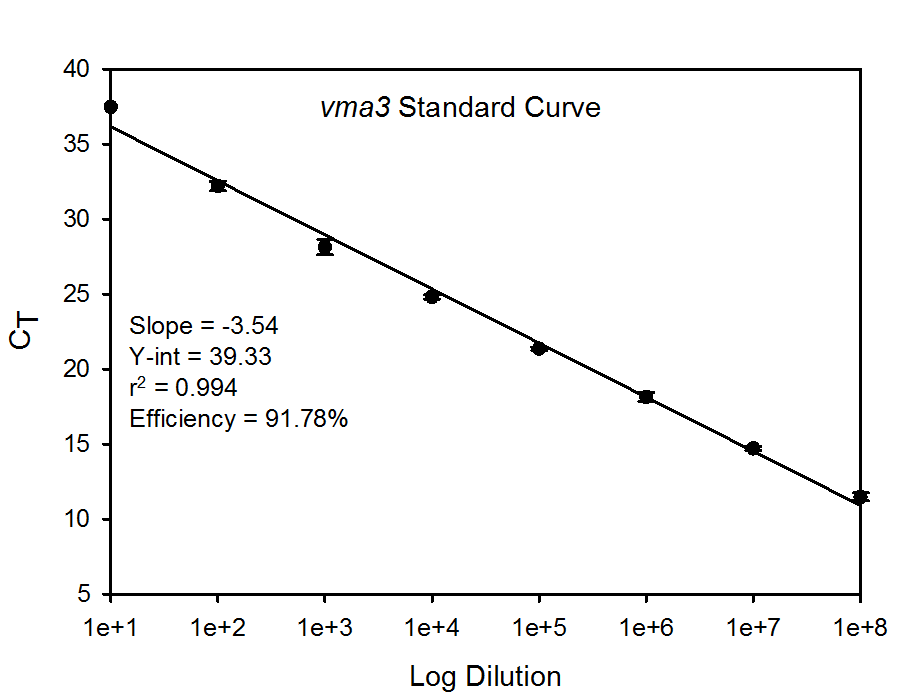

Supplement: Figure S4 — Standard curve of vma3 assay, constructed as described for the act assay. (TIF) [file pone.0112706.s004.tif]

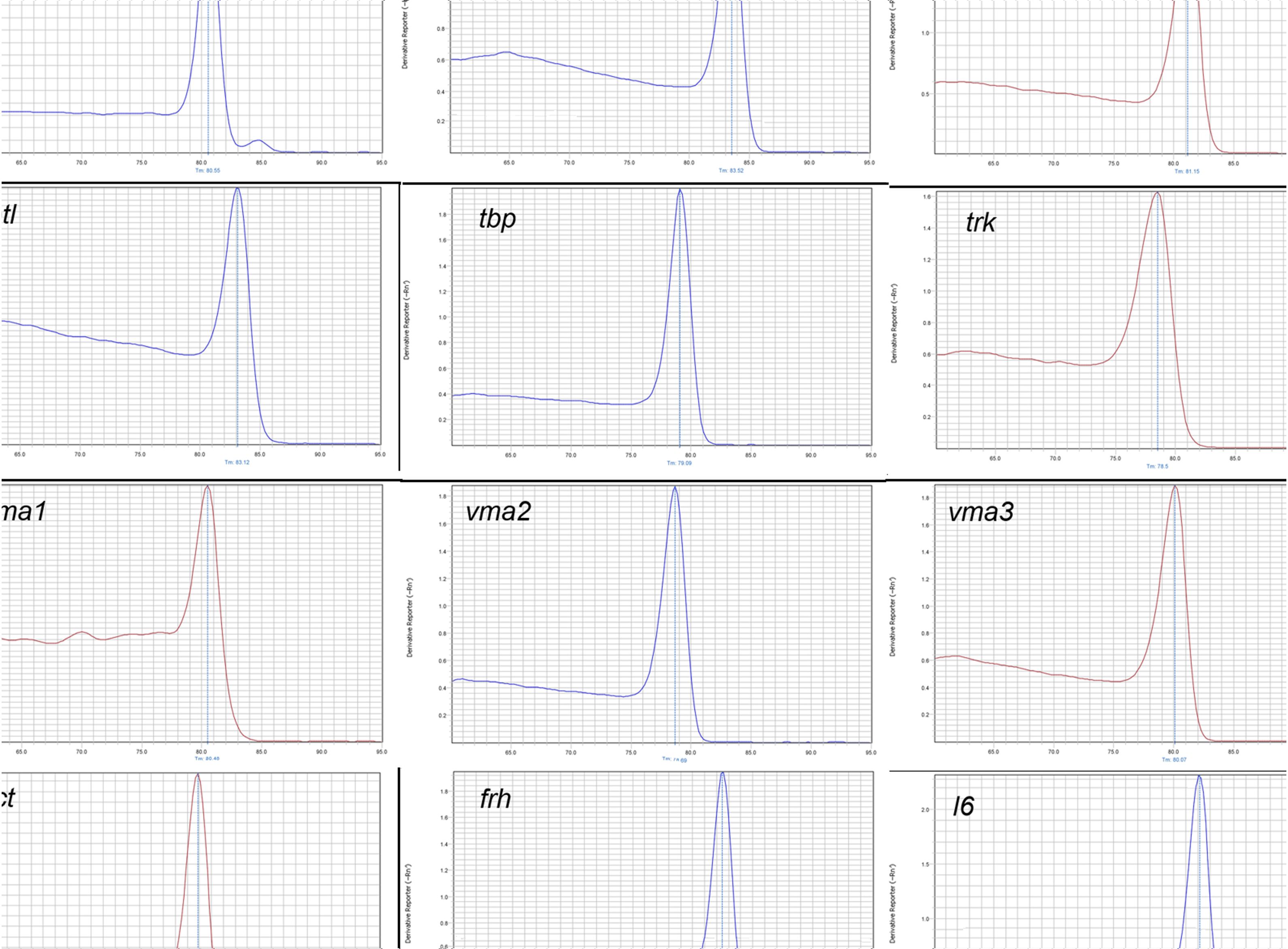

Supplement: Figure S5 — Melt curve profiles of the 12 reference gene assays evaluated in this study. (TIF) [file pone.0112706.s005.tif]

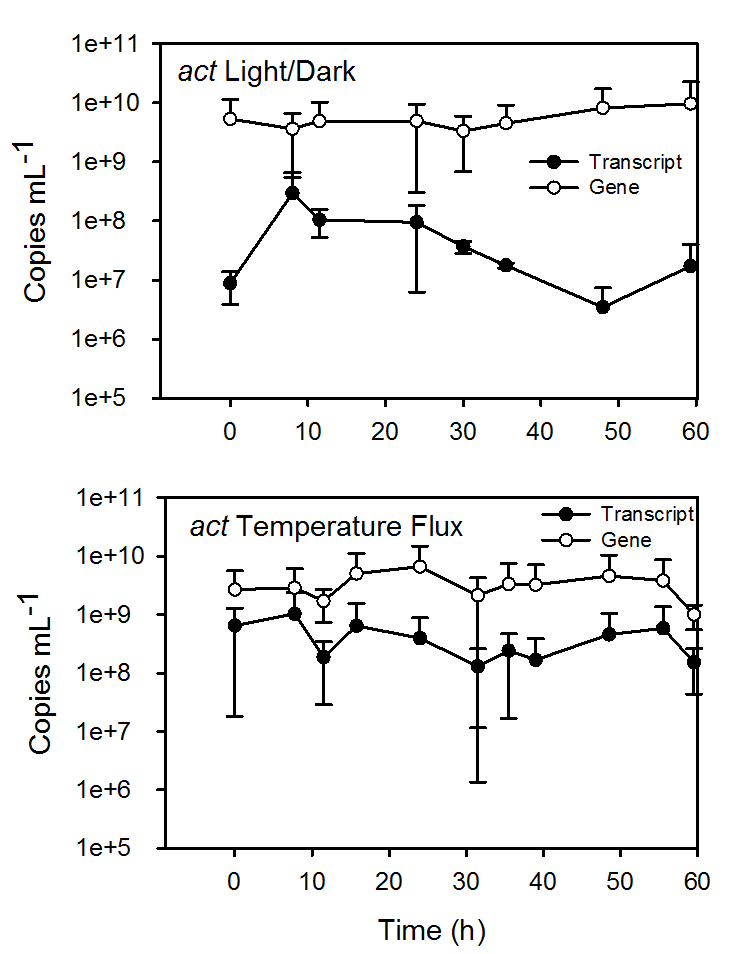

Supplement: Figure S6 — act transcript and gene copies over time under conditions of light/dark cycling, temperature flux, and continuous darkness. Transcript and gene copies per mL of sample were calculated via absolute quantification using external calibration curves as described in the text. Error bars represent the standard deviation derived from triplicate qPCRs from biological replicates. (TIF) [file pone.0112706.s006.tif]

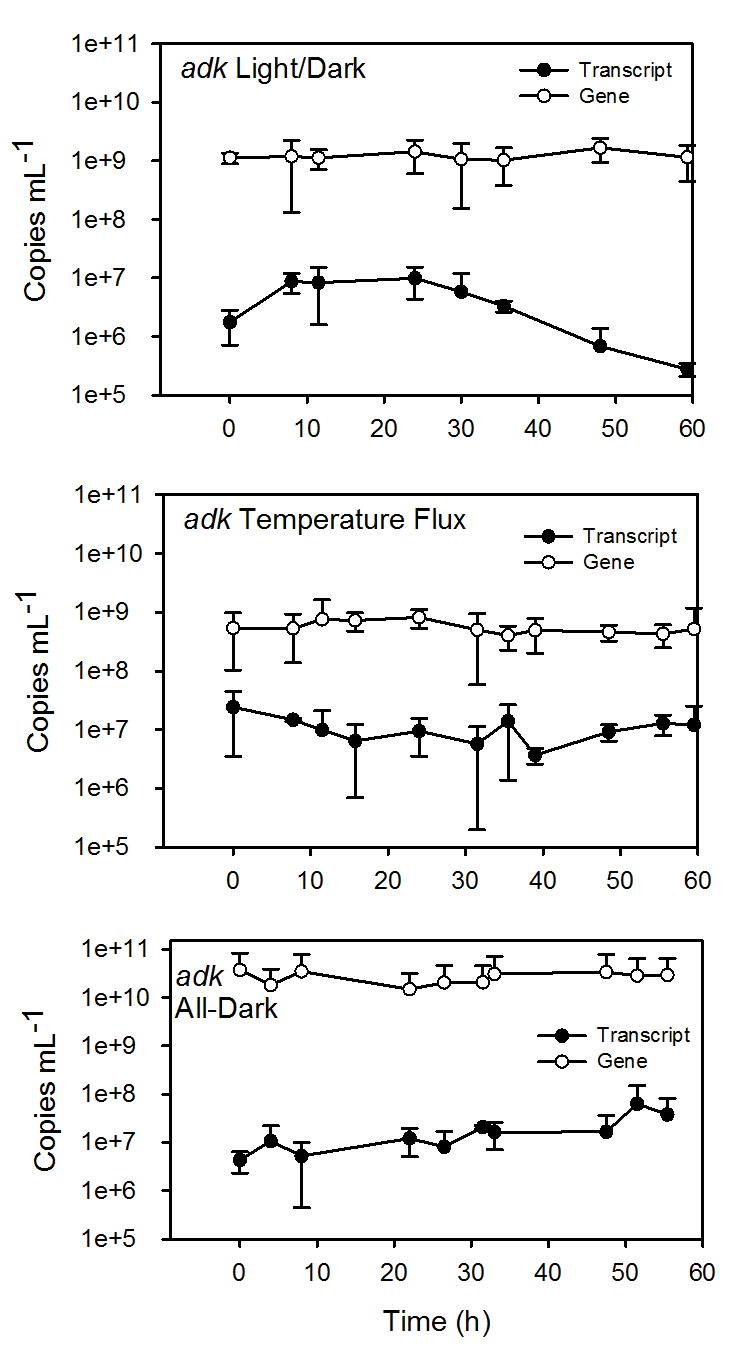

Supplement: Figure S7 — adk transcript and gene copies over time under conditions of light/dark cycling, temperature flux, and continuous darkness. Transcript and gene copies per mL of sample were calculated via absolute quantification using external calibration curves as described in the text. Error bars represent the standard deviation derived from triplicate qPCRs from biological replicates. (TIF) [file pone.0112706.s007.tif]

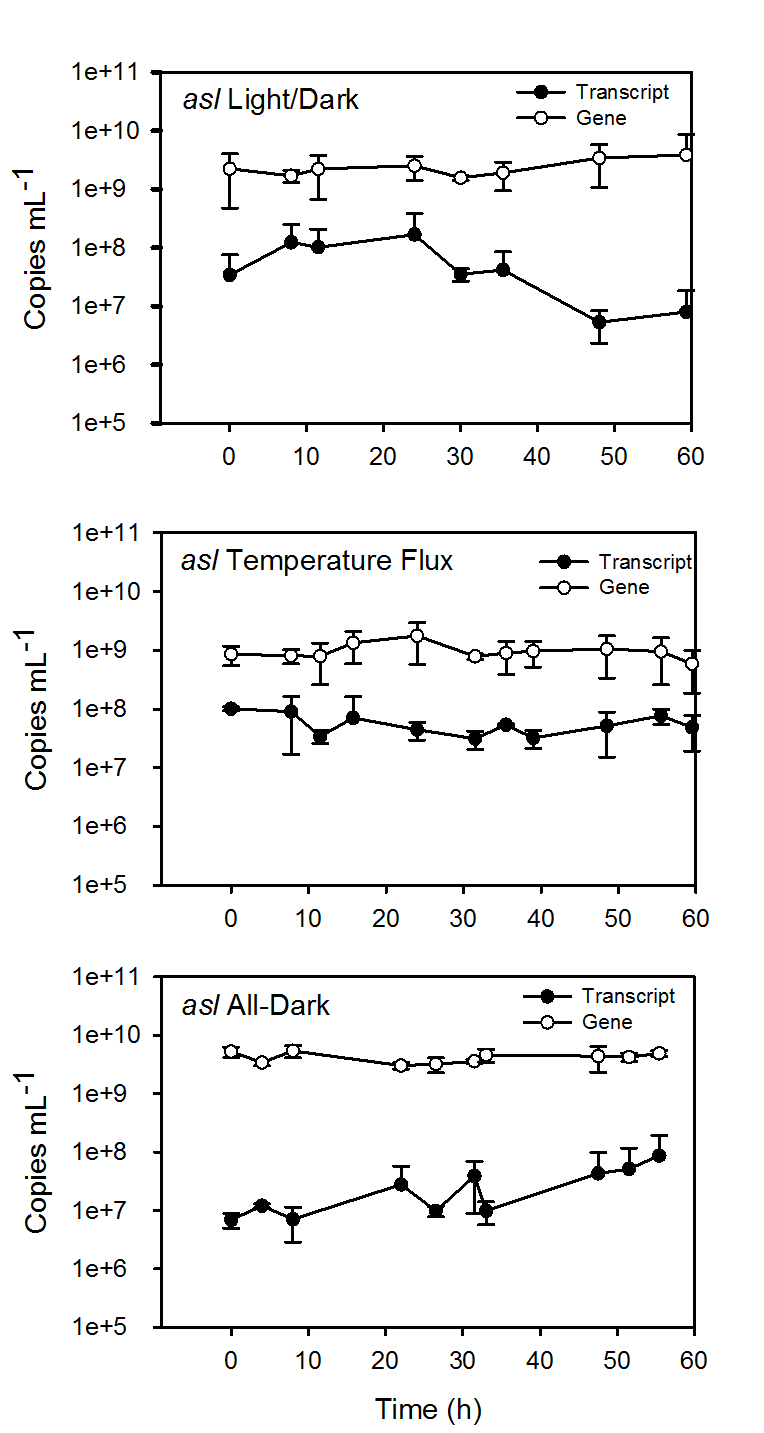

Supplement: Figure S8 — asl transcript and gene copies over time under conditions of light/dark cycling, temperature flux, and continuous darkness. Transcript and gene copies per mL of sample were calculated via absolute quantification using external calibration curves as described in the text. Error bars represent the standard deviation derived from triplicate qPCRs from biological replicates. (TIF) [file pone.0112706.s008.tif]

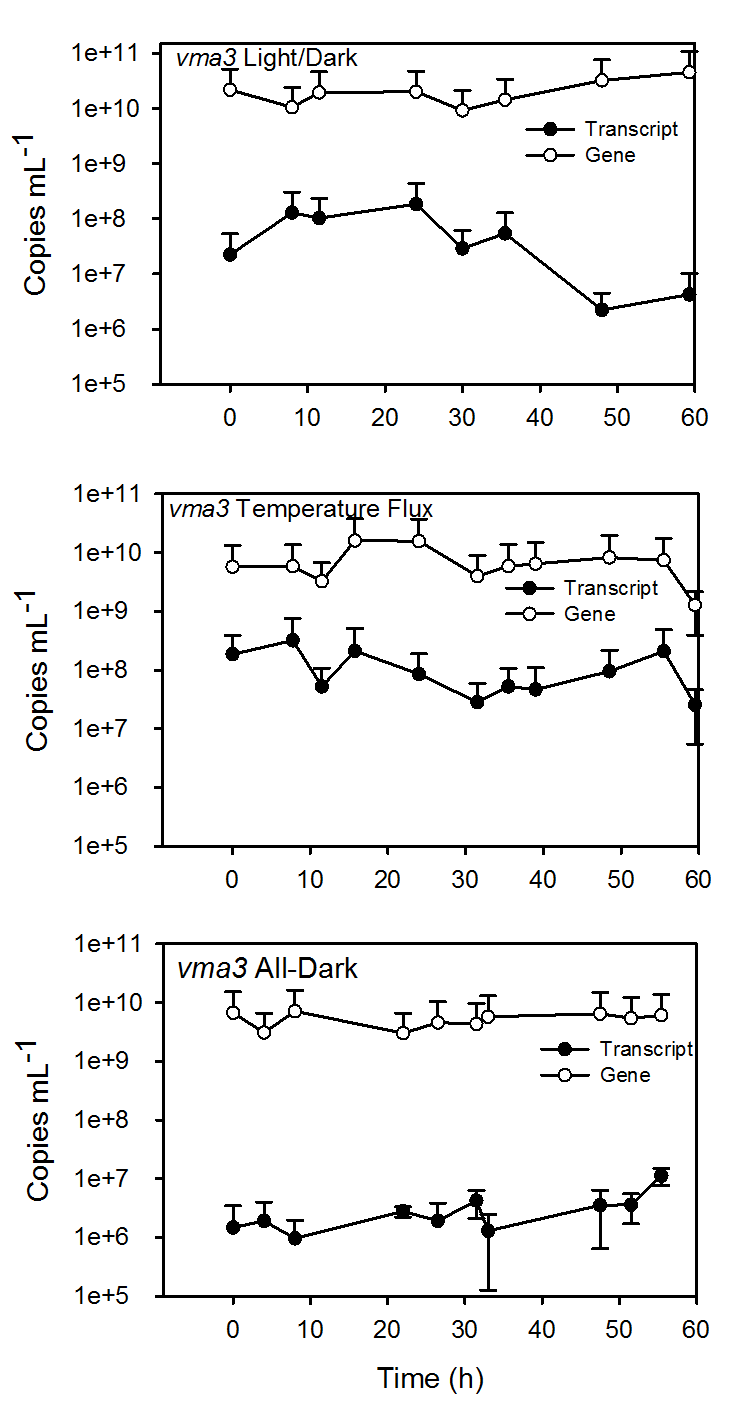

Supplement: Figure S9 — vma3 transcript and gene copies over time under conditions of light/dark cycling, temperature flux, and continuous darkness. Transcript and gene copies per mL of sample were calculated via absolute quantification using external calibration curves as described in the text. Error bars represent the standard deviation derived from triplicate qPCRs from biological replicates. (TIF) [file pone.0112706.s009.tif]
