# Supplementary material for: Selection and Evaluation of Reference Genes for Expression Studies with Quantitative PCR in the Model Fungus Neurospora crassa under Different Environmental Conditions in Continuous Culture
Source: PLoS One. 2014 Dec 4;9(12):e112706. doi: 10.1371/journal.pone.0112706 (PMC4256298; doi:10.1371/journal.pone.0112706)
Supplement: Table S1 — Primers used in construction of act , adk , asl , and vma3 external standards for absolute quantification. (PDF) [file pone.0112706.s010.pdf]

Gene	Primer Sequence (5'-3')
ACT	TCTTCTTCTGTACCTTTTGCT
	CTTCTCCTTGATGTCACGAA
ADK	CTTTGGCGATGAGGCTCTC
	CTCCTGGATGGAGAGCTTA
ASL	CTTCGCGACTCTCCGTGA
	GAAGCGAGGATGGCATCC
VMA3	CCCTGTTTACGCGGTTAG
	CAAGACTTCGGCGAAAATG
