# Supplementary material for: Selection and Evaluation of Reference Genes for Expression Studies with Quantitative PCR in the Model Fungus Neurospora crassa under Different Environmental Conditions in Continuous Culture
Source: PLoS One. 2014 Dec 4;9(12):e112706. doi: 10.1371/journal.pone.0112706 (PMC4256298; doi:10.1371/journal.pone.0112706)
Supplement: Table S2 — Normality assessment of transcript: gene ratio and CT values of act , adk , asl , and vma3 genes by Shapiro-Wilkes test. (PDF) [file pone.0112706.s011.pdf]

Table S2.

Gene/Condition	Ratio W	Ratio p	Pass/Fail	C_T W	C_T p	Pass/Fail
**ACT Light/Dark**	0.829	0.058	Passed	0.915	0.388	Passed
**ACT All Dark**	0.847	0.054	Passed	0.977	0.950	Passed
**ACT Temp**	0.842	0.029	Failed	0.957	0.741	Passed
**ASL Light/Dark**	0.916	0.402	Passed	0.88	0.188	Passed
**ASL All Dark**	0.887	0.157	Passed	0.924	0.395	Passed
**ASL Temp**	0.896	0.141	Passed	0.948	0.601	Passed
**ADK Light/Dark**	0.909	0.349	Passed	0.908	0.339	Passed
**ADK All Dark**	0.761	0.005	Failed	0.934	0.489	Passed
**ADK Temp**	0.862	0.052	Passed	0.924	0.324	Passed
**VMA3 Light/Dark**	0.922	0.488	Passed	0.887	0.219	Passed
**VMA3 All Dark**	0.838	0.042	Failed	0.934	0.490	Passed
**VMA3 Temp**	0.841	0.028	Failed	0.986	0.997	Passed
